# Update on retinal vascular caliber

**DOI:** 10.22336/rjo.2017.32

**Published:** 2017

**Authors:** Alina Gabriela Dumitrescu, Liliana Voinea, Ioana Anca Badarau, Vanessa Andrada Paun, Marilena Schowe, Radu Ciuluvica

**Affiliations:** * Coltea Clinical Hospital, Bucharest, Romania; ** Physiology Department I, “Carol Davila” University of Medicine and Pharmacy, Bucharest, Romania; *** Ophthalmology Department, University Emergency Hospital; “Carol Davila” University of Medicine and Pharmacy, Bucharest, Romania; **** Ophthalmology Hospital, Langenfeld, Dortmund, Germany; ***** Anatomy Department, “Carol Davila” University of Medicine and Pharmacy, Bucharest, Romania

**Keywords:** retinal vascular caliber, vessel diameter, hypertension, diabetes, optical coherence tomography, fundus photography

## Abstract

Retinal vessels are the only blood vessels that can be viewed directly, in vivo, repetitively and non-invasively. Retinal vessel caliber is influenced by physiological (age, sex, race, blood pressure, blood glucose, body mass index) and pathological (atherosclerosis, dyslipidemia, smoking) determinants. There are studies on large population groups that demonstrate the associations between retinal vasculature and subclinical or clinical changes in systemic diseases (hypertension, diabetes, stroke, renal or cardiac diseases). The assessment of retinal vessels can provide information about the pathophysiology of many diseases, but it also has a direct applicability in clinic, being used as a screening method that predicts the risk of their occurrence.

## Introduction

The development of ophthalmoscopy in the 19th century has allowed the in vivo, non-invasive study of the retina and its microcirculation. Thus, numerous correlations have been made between changes in the appearance of retinal vessels and the various systemic diseases involving vascular alterations. The most frequent changes in retinal vasculature were observed in hypertensive patients, attempting to make a classification for hypertensive retinopathy. There are several such classifications, but the most well-known and used is that of Keith from 1974 [**1**].

**Table 1:** Keith-Wagner-Barker classification of hypertensive retinopathy

Grade	Retinal vessel morphology
I	Mild to moderate narrowing or sclerosis of the arterioles
II	Moderated to marked arteriolar sclerosis, arteriovenous nicking, generalized and/ or focal arteriolar narrowing
III	Marked arteriolar narrowing and focal constriction, retinal edema, cotton wool spots, hemorrhage
IV	Grade III plus papilledema

The disadvantage of this method is the degree of subjectivity and variability among examiners, as well as the difficulty of an objective assessment of incipient changes that may allow the early therapeutic interventions able to reduce the risk of long-term complications.

The emergence of new retinal imaging methods, such as retinal photographs and optical coherence tomography, allowed a more objective and reproducible assessment of the vascular caliber. Hubbard and collaborators have developed computational formulas for CRAE (central retinal arteriolar equivalent) and CRVE (central retinal venular equivalent) through retinal photography [**2**]. Based on these coefficients, the AVR (arteriovenous ratio) used in many studies is calculated because its value is not dependent on the optical properties of the eye or the camera. But, this method also has limitations because it does not allow a direct measurement of the vascular caliber, it is based on a single image and does not provide information about the three-dimensional geometry of the vessel [**3**]. The appearance of OCT (optical coherence tomography) offers three-dimensional images of vessels, easy to obtain and independent of magnification. Thus, Goldenberg used a horizontal cube scan at the optic disc margin for retinal vessel diameter measurement with SD-OCT (spectral domain OCT) [**4**]. Moreover, Muraoka used a circular scan to detect the cross section image of the retinal vessel and the measured retinal vessel diameter, the lumen diameter and the wall thickness in zone A [**5**] and Zhu used a line scan perpendicular to the vessel to measure the vessel diameter and the lumen diameter in zone B [**6**].

There are new vascular parameters that began to be measured in relation to the cardiovascular changes. These are the branching angle of vessels, retinal vessel tortuosity, and fractal dimensions. Thus, the local topographic features of retinal vessels may provide information on the appearance of systemic and cerebral microcirculation. The development of new technologies will allow the dynamic and functional examination of the retinal vasculature by the flickering light response used by the Doppler flowmeter and the dynamic vessel analyzer [**7**,**8**]. There is still insufficient data on the information provided by these innovative methods.

With all these methods available, studies have been conducted on large groups of individuals to find correlations between the retinal vessel diameter and the demographic characteristics and the associated systemic pathology of people.

**Determinants of retinal vascular caliber**

Age

A first study on 20 newborns reported an average value of 85.5 μm retinal arterioles and a 130 μm retinal diameter without gender differences [**9**]. In the first 6 years of life, when the body weight increases about 5-fold, the arteriolar and retinal caliber doubles [**10**]. By adulthood, the venular diameter remains constant, while the arteriolar caliber continues to increase [**11**]. Then, after the age of 45, both the arteriolar and the venular caliber begin to slowly decrease by 1.8-4.8 μm for each decade of age [**12**]. Therefore, by the age of 80 years, the vascular caliber is with 10-12 μm, smaller than at 55-60 years [**13**].

Sex

In the Cardiovascular Health Study group and MESA (The Multi-Ethnic Study of Atherosclerosis), higher values of retinal arteriolar caliber and AVR were found in women compared to men, linking these values to the protective effect of estrogens on coronary circulation [**14**,**15**].

Race

Both arteriolar and venular caliber have higher values in African-American and Hispanic races than in Caucasians and Chinese. These values may correlate with the differences in the distribution of cardiovascular risk factors among populations of different ethnicity, but the data are insufficient [**15**,**16**].

Birth weight

Mitchell measured the retinal vascular diameter in a study on 1,369 children and estimated that the average arteriolar diameter was lower by 2 μm for every kilo in minus to the birth weight [**10**]. A twin study in Tasmania found a direct correlation between the birth weight and the retinal arteriolar caliber [**17**]. These changes may suggest a structural adaptation of microcirculation to nutrition and growth restriction. Also, children with a small weight at birth are at a greater risk of developing a cardiovascular disease in adulthood.

Genetics

Data from the Beaver Dam Eye Study, based on genome analysis, demonstrates a genetic contribution to the variation of the retinal vasculature, with similar values for individuals in the same family [**18**]. In another large cohort study, four genes responsible for retinal vasculature variation (19q13, 6q24, 12q24, and 5q14) were identified. In nearby regions, genes for essential hypertension, coronary heart disease, endothelial dysfunction, and angiogenesis are located [**19**]. These new data suggest the importance of studying retinal microcirculation to understand the pathogenesis of the cardiovascular disease.

**Modifiable factors that influence retinal vasculature**

Blood pressure (BP)

There is a clear association between hypertension and peripheral arterial narrowing [**20**]. Initially, it was believed that the impaired sodium homeostasis is responsible for the fluid expansion that leads to cardiac overload and the BP increase with secondary structural changes on peripheral circulation [**21**]. New hypotheses consider the arterial changes responsible for arterial hypertension [**22**]. Studies on laboratory animals have shown decreases in retinal arteriolar diameters prior to arterial hypertension in rats [**23**] and studies in humans showed that people with a family history of hypertension have lower arteriolar diameters than those with no history [**24**].

Blood glucose 

Many studies have correlated the presence of hyperglycemia with a higher retinal vein diameter [**25**]. Hyperglycemia and secondary retinal ischemia increase blood flow, while altered glucose metabolism generates inflammatory cytokine and nitric oxide release, processes leading to endothelial dysfunction with secondary venular dilation [**26**]. 

Body mass index (BMI)

Studies such as MESA and ARIC (The Atherosclerosis Risk in Communities Study) have found an association between the increased retinal vein caliber and the obesity indicators (high body mass index and increased waist-hip ratio). The Blue Mountain Eye Study reports that an increased retinal diameter may predict the incidence of obesity in 5 years, suggesting that the microcirculation dysfunction is involved in the pathogenesis of obesity [**27**]. The Singapore Prospective Study Program also found a link between the lack of physical activity and the larger retinal venular caliber [**28**].

Dyslipidemia

Both, MESA and ARIC have demonstrated a correlation between the increased retinal venular caliber and dyslipidemia defined as elevated plasma levels of triglycerides and LDL cholesterol and low HDL cholesterol [**29**,**30**].

*Atherosclerosis*

A link between low AVR and the presence of carotid artery atheromatosis was demonstrated in the ARIC study [**16**]. Moreover, the Rotterdam study correlated the small AVR with the intima-mean thickness of the carotid artery wall as well as the increased stiffness of the arterial wall (marker of atherosclerosis) [**29**]. MESA related a retinal arterial narrowing with a concentric remodeling of the left ventricle and the myocardial blood flow, while the larger venous caliber is associated with the low index ankle arm [**31**,**32**].

In Beaver Dam Eye Study, the indicators of endothelial dysfunction (soluble intercellular adhesion molecule-1 and E-selectin serum) and the inflammatory markers (PCR and leukocyte counts) were associated with an increased retinal venous diameter [**33**]. Thus, atherosclerosis, inflammation, and endothelial dysfunction play an important role in demonstrating the link between the retinal venular dilatation and the systemic cardiovascular disease.

Smoking

The ARIC study demonstrated an association between low AVR and cigarette smoking in middle-aged individuals. In the Rotterdam study, higher values of arteriolar and retinal venular caliber were correlated with smoking [**16**,**29**].

Alcohol

Recent studies have linked alcohol consumption to the risk of cardiovascular disease, but few have also analyzed the relationship with retinal vasculature [**16**]. The ARIC study found lower AVR values for alcohol consumers and MESA narrower arteriolar caliber [**14**]. Longitudinal studies are required to establish the mechanism by which alcohol consumption influences the retinal vascular size.

Drug administration

The Beaver Dam Eye Study reported an association between the topical beta blocking agents for the treatment of glaucoma and the narrow retinal arterioles and venules; Beta blockers administered orally do not have the same effect [**34**]. According to the Blue Mountain Eye Study, retinal vascular diameters remain constant over time, suggesting the protective effect of estrogens on the long term in women who receive substitution hormone therapy for more than 10 years [**35**]. This study also reported a possible association between the combined aspirin-antihypertensive therapy and the larger diameters of retinal arterioles, which confirms the anti-inflammatory effect of aspirin on microcirculation [**36**].

**Associations between retinal vasculature and systemic diseases**

Hypertension

It is well known that the narrowing of the retinal arteriolar caliber is an early sign of hypertension secondary to chronic exposure to high blood pressure. A meta-analysis that comprised 5 large studies with a total of 19,633 patients reported that for every 10 mmHg of BP increase there was a decrease of 2.0-2.4 μm of the retinal arteriolar caliber [**37**]. The Rotterdam study reported a greater influence of BP on the retinal vascular size in younger and more prominent on arterioles than venules [**22**]. This hypothesis can be supported by the fact that, in the elderly, the stiffness and the arterial wall sclerosis prevent vascular adaptation to higher blood pressures. Thus, the narrow arteriolar caliber may be a marker for the assessment of the chronic effects of hypertension (aortic wall stiffness, hypertrophy and remodeling of the left ventricle) [**38**]. Beaver Dam, ARIC, Blue Mountains, and Rotterdam have found lower values of retinal arteriolar diameter and AVR in patients who subsequently developed hypertension in 3 years than in those with a normal blood pressure. These new data make the arteriolar caliber and AVR a pre-clinical marker of hypertension. A raised peripheral vascular resistance in small vessels, including retinal vessels, is the initial condition for the development of essential hypertension [**22**,**39**].

Diabetes Mellitus

Microvascular pathology has an important role in the pathophysiology of diabetes mellitus [**40**]. Since the retinal vessels are the only small vessels that can be readily available, we can study the effect of hyperglycemia on the retinal vascular caliber [**41**]. Some studies (ARIC and Beaver Dam Eye) showed a link between the low AVR and the higher risk of developing Diabetes Mellitus independent of the cardiovascular risk factors [**42**]. In the Rotterdam Eye Study, the increased venular diameter was correlated with the impaired fasting glucose. In subsequent studies (MESA, Australian Diabetes, Obesity and Lifestyle and Blue Mountains Eye Study), an association was also reported between Diabetes and the increased retinal arteriolar caliber [**43**-**45**]. In WESDR (Wisconsin Epidemiologic Study of Diabetic Retinopathy), in patients with type 1 diabetes mellitus, a higher glycosylated hemoglobin was associated with a higher retinal vascular caliber, both venules and arterioles [**46**]. 

Coronary heart disease

Studies have found an association between the retinal microvascular morphology and the incidence of coronary pathology [**47**]. The ARIC study correlated a small AVR with the high risk of coronary artery disease in women, and CHS demonstrated that small AVR is generated by both, narrow arteriolar caliber and increased venular caliber [**48**]. The Blue Mountains Eye Study and the Beaver Dam Eye study correlated small AVR (due to larger venular caliber) with the double risk of coronary heart disease mortality in persons under 75 years old. For women, an additional 1.5-fold increase in risk is associated with a narrow arteriolar caliber [**49**]. In his meta-analysis of 22,159 people, McGeechan revealed a correlation between the narrow arteries and the larger venules and the risk for coronary artery disease in women [**50**]. In WESDR, in patients with type I diabetes mellitus, the risk of cardiovascular mortality is higher in those with low AVR. In some studies, the smaller retinal arteriolar caliber was associated with signs of concentric remodeling of the left ventricle, seen on magnetic resonance imaging (MRI). Thus, the retinal microvascular changes may provide information on subclinical myocardial abnormalities, especially in middle-aged women (49-75 years) [**15**,**31**,**46**].

Brain diseases

It has been hypothesized that the impaired cerebral microcirculation is a major risk factor for stroke [**51**]. Since small cerebral vessels are quite similar in embryological origin, structure and function to retinal vessels, an attempt is being made to link the appearance of retinal vessels with brain pathologies [**52**]. Cardiovascular Health Study (CHS) and ARIC reported an association between an increased risk of ischemic stroke and low AVR; Rotterdam study demonstrated that people with a larger retinal venular caliber have a 12% higher risk of developing cerebral infarction [**53**]. McGeechan also concluded that the stroke risk is associated with an increased retinal venular diameter and there is no correlation with the arteriolar caliber [**54**]. There is only one study demonstrating the correlation between the increased venular caliber and the risk of intracerebral hemorrhage [**55**]. In WESDR, in patients with type II diabetes mellitus, both narrow arterioles and the dilated venules are correlated with a high mortality caused by stroke after 22 years [**46**]. Also, in ARIC, CHS and Rotterdam Study, subclinical changes in MRI (lacunar infarcts, white matter lesions and cortical atrophy) were found in healthy individuals with a larger venular caliber [**56**,**57**]. Patients without stroke retinal venular changes were associated with a poorer cognitive function and an increased risk of developing vascular dementia [**58**]. In conclusion, there are numerous links between the retinal microvascular changes and clinical cerebral pathologies, such as stroke, or sub-clinical, respectively, small vessels disease.

Kidney diseases

There are fewer studies on kidney diseases and vascular morphology, many of them on groups of patients with diabetes, in whom retinopathy and nephropathy are thought to have a common pathogenic pathway (endothelial dysfunction due to inflammation) [**59**,**60**]. WESDR demonstrated that in patients with type I Diabetes, the larger retinal venular diameter predicted proteinuria and renal impairment on the long run, and in those with type II diabetes, the venular retinal dilatation occurred before nephropathy [**61**]. ARIC revealed that low AVR was correlated with higher serum creatinine levels in both diabetic and non-diabetic patients [**60**]. In a study from 2017, Daien concluded that both CRAE and CRVE were negatively correlated with urinary albumin excretion and did not correlate with the estimated glomerular filtration rate [**62**]. Further, studies are needed to demonstrate correlations between retinal microvascular changes and renal dysfunctions. It is known that microvascular changes are important in the pathophysiology of diabetic nephropathy [**60**].

**Ocular diseases**

In the pathophysiology of many ocular diseases, changes in retinal vessels are caused by cardiovascular pathologies.

Diabetic retinopathy

The MESA study, conducted on approximately 6,000 people of different ethnicities with normal blood sugar, hyperglycemia or diagnosed with Diabetes, evaluated retinal microcirculation. Thus, higher CRAE values were found in patients with Diabetes while CRVE was higher in those with impaired fasting glucose and those with Diabetes. Only large venous caliber was correlated with changes in glycemic and glycosylated hemoglobin, as well as the presence of diabetic retinopathy [**44**]. The combination of increased venular diameters with diabetes was also demonstrated by ARIC, Rotterdam and WESDR [**63**]. The WESDR study on patients with type II diabetes mellitus found an association between small CRAE and the increased risk of lower limb amputations and stroke mortality. Large CRVE was associated with an increased incidence of nephropathy and stroke mortality. Also in WESDR, large venular caliber at the onset of diabetes was associated with a higher incidence of proliferative diabetic retinopathy on the long run [**61**]. According to the Diabetic Retinopathy Study, the increase in the retinal venular caliber is a predictor of a low visual acuity due to the progression of diabetic retinopathy. Arterial and venular changes may be caused by the presence of endothelial dysfunction due to the inflammation and retinal hypoxia caused by hyperglycemia [**64**,**65**]. 

Age-related macular degeneration (AMD)

Epidemiological studies have shown that both AMD and the cardiovascular disease have similar risk factors (smoking, high blood pressure, atherosclerosis), as well as an increased risk of stroke and myocardial infarction [**66**]. Due to these data, hypotheses were made that the pathological processes involved in the occurrence of AMD may lead to changes in the retinal vasculature. Longitudinal studies on Caucasians, Beaver Dam Eye Study, Blue Mountains Eye Study (BMES), and Rotterdam Study have found no association between the retinal vascular caliber and AMD [**67**]. A study on the Asian population, the Singapore Malay Eye Study, concluded that a higher retinal venular diameter is associated with retinal pigment abnormalities and early stage AMD. Although a smaller arterial diameter was associated with retinal pigmentation abnormalities, there is no significant correlation between arteriolar caliber and AMD occurrence [**68**]. In contrast, the Handan Eye Study showed that a narrow arteriolar diameter is associated with incipient forms of AMD, while the venular caliber is not [**69**]. 

Primary Open-Angle Glaucoma (POAG)

Glaucomatous optic neuropathy is defined by reduced retinal nerve fiber layer (RNFL) thickness, increased Cup Disc ratio and characteristic visual field defects [**70**]. There are many hypotheses regarding the correlations between the retinal arteriolar caliber reduction and RNFL loss. These hypotheses are supported by the fact that the loss of retinal ganglion cells decreases local metabolic and vascular requirements, leading to arteriolar vasoconstriction [**71**]. Various study hypotheses have been outlined. In a group of patients with asymmetric glaucoma, De Leon found lower values of CRAE and CRVE in the eye with severe glaucomatous impairment [**72**]. In BMES, lower arteriolar caliber values were found in POAG patients than in non-glaucoma patients [**73**]. The Singapore Malay Eye study found significant associations between the retinal vascular size and glaucoma prevalence as well as a larger cup disc ratio [**74**]. The Beijing Eye Study showed that POAG patients had a smaller retinal arteriolar caliber and a similar venular caliber compared to those without glaucoma [**75**]. In the Handan Eye Study, both POAG and PCAG (primary closed angle glaucoma) patients have a retinal arteriolar and a retinal venular caliber smaller than the non-glaucoma control group [**76**]. Hall found a significant correlation between the retinal arteriolar diameter and the visual field defects in POAG patients [**77**]. Both Beaver Dam Eye Study and Rotterdam Study have not found correlations between the retinal vascular caliber and glaucoma prevalence, increased Cup Disc ratio or high IOP. Further studies are needed to define the role of retinal vasculature in the pathogenesis of POAG.

*Retinal vein occlusion*

There was only one study in Korea, on 10890 people, of which 0.8% were diagnosed with branch retinal vein occlusion, which measured CRAE and CRVE. The results showed that BRVO eyes had lower mean CRAE and CRVE values compared to normal eyes [**78**].

## Conclusions

In conclusion, there are links between retinal vasculature and the large number of physiological or pathological changes in the human body. Narrow retinal arteries and, possibly, a small AVR are found in elderly, hypertensive, with vascular atherosclerosis and alcohol-consuming patients. Moreover, patients with POAG have a small arteriolar caliber. There are cases of increased arteriolar diameter, such as females (possibly because of hormone replacement therapy), African-American or Hispanic race, smokers, or people who have antihypertensive and aspirin-associated treatment. An increased retinal venular caliber is found in African-American and Hispanic patients, in those with a large BMI, dyslipidemia, atherosclerosis, who smoke, have an impaired fasting glucose, or diabetes and with cerebrovascular disease (stroke or vascular dementia). In patients with diabetes mellitus, a larger venous caliber suggests an increased risk of retinopathy and diabetic nephropathy. Combined large venular and narrow arteriolar caliber can predict an increased risk of coronary artery disease, especially in women, or may suggest a higher mortality caused by stroke in patients with Diabetes.

Retina examination offers information about many systemic disorders. The fact that it can be explored directly, in vivo and noninvasively, makes eye examination a mandatory investigation in all patients. The new imaging techniques of the last decade allow an easy exploration of retinal circulation. Retinal vascular caliber is a quantitative assessment of microvascular structural changes that can be correlated with the cardiovascular risk factors or the glycemic profile. Numerous studies have demonstrated that the value of retinal vascular caliber can also be used as a noninvasive biomarker for the most important 21st century diseases (cardiovascular disease and diabetes). More and more rapid and easy examination methods of retinal vasculature will be developed and used in the coming years.

**Conflict of interests**

The authors declare no conflict of interest.
